# Vitamin B6 Toxicity Secondary to Daily Multivitamin Use: A Case Report

**DOI:** 10.7759/cureus.48792

**Published:** 2023-11-14

**Authors:** Alexander Paluszny, Shuhao Qiu

**Affiliations:** 1 Internal Medicine, The University of Toledo Medical Center, Toledo, USA

**Keywords:** multivitamin supplement, vitamin b6 supplementation, multivitamin toxicity, vitamin b6 toxicity, peripheral neuropathy, pyridoxine toxicity, vitamin b6

## Abstract

Vitamin B6 is a water-soluble vitamin that is an important cofactor in various metabolic processes. Although rare, its consumption can sometimes result in toxicity, which typically presents with peripheral neuropathy in the early stage. While vitamin B6 toxicity is most often associated with supplemental mega-doses of more than 50 mg/day, more recent studies have shown that toxicity can occur in cases of much smaller doses as well. We present a case of a 73-year-old male with a three-year history of progressive peripheral neuropathy who was found to have a serum vitamin B6 level of 259.9 nmol/L (reference range: 20-125 nmol/L) but only reported taking a daily multivitamin containing 6 mg of vitamin B6. This case of toxicity in the setting of a daily intake lower than the European Food Safety Administration's (EFSA) newly established Tolerable Upper Intake Level (UL) of 12 mg/day highlights the need for further research into the effects of relatively low-dose vitamin B6 supplementation.

## Introduction

Vitamin B6 is a water-soluble vitamin that plays a role in multiple enzymatic reactions critical for the metabolism of carbohydrates, lipids, and amino acids. The recommended daily intake of vitamin B6 is between 1.3-2.0 mg/day, which can be found in foods such as poultry, fish, potatoes, fortified grains, and fruit [1]. As an essential nutrient, vitamin B6 is also found in dietary supplements, such as multivitamins, although dietary deficiency is rare. Intake of food-based sources of vitamin B6 will not cause toxicity, but the use of supplements containing the nutrient potentially can, classically presenting with peripheral neuropathy [2]. The less commonly reported symptoms include ataxia, muscle weakness, disequilibrium, and dermatosis [3]. As one of the rarer causes of peripheral neuropathy, vitamin B6 toxicity can often be overlooked, leading to a delay in diagnosis. We present a patient with a three-year history of peripheral neuropathy secondary to vitamin B6 toxicity due to daily multivitamin use.

## Case presentation

A 73-year-old male presented to his primary care physician for a wellness visit with a three-year history of peripheral neuropathy. He initially reported numbness in the bilateral lower extremity, usually only at night. The patient denied any back pain or weakness in his legs. The physical exam showed normal pulses, strength, reflexes, gait, and mental status with decreased vibratory sense on the left ankle and decreased monofilament sensation bilaterally. Preliminary laboratory evaluation revealed vitamin B12, thyroid levels, HbA1C, and antinuclear antibodies to be within the reference range. A previous peripheral neuropathy workup from 2020 was also unremarkable.

The patient was seen in the clinic again eight months later with similar complaints of progressive bilateral numbness and tingling in the lower extremities with occasional involvement of his fingertips. The physical exam again showed normal pulses, strength, reflexes, gait, and mental status with decreased vibratory sense bilaterally. The patient was referred for electromyography at this time, which showed bilateral sensory neuropathy of unknown etiology with early signs of progression to motor fibers. The patient was instructed to follow up with his primary care physician for further evaluation.

At this follow-up visit, the patient reported new-onset lightheadedness and dizziness. Repeat laboratory evaluations were completed, including serum vitamin B6, which was found to be 259.9 nmol/L (reference range: 20-125 nmol/L). The patient had been taking a daily multivitamin containing 6 mg of vitamin B6 for more than 10 years, which he was instructed to discontinue at this time. When asked about his diet, the patient reported eating a balanced diet and denied any recent changes. He denied taking any additional vitamins, energy drinks, workout supplements, or other items with known high amounts of vitamin B6.

One month after stopping the multivitamin, the patient reported relief from his dizziness and a slight improvement in his numbness and tingling. He was advised to continue to monitor his neuropathy symptoms and follow up with repeat serum vitamin B6 in four months.

## Discussion

The Recommended Dietary Allowance (RDA) of vitamin B6 varies slightly depending on age and gender. The RDA is 1.0 mg/day for children aged 9-13 years, 1.2 mg/day for girls aged 14-18 years, and 1.3 mg/day for boys aged 14-18 years. For adults, the RDA is 1.3 mg/day for those aged 19-50 years, 1.5 mg/day for women older than 50 years, and 1.7 mg/day for men older than 50 years. The estimated dietary intake in the US is 1.5 mg/day for women and 2.0 mg/day for men [1]. The multivitamin that our patient reported taking daily contained 6 mg of vitamin B6, more than 350% of the RDA for older men. However, this is still seen as a relatively low dose of vitamin B6 supplementation as products such as energy drinks can have up to 40 mg of vitamin B6, 2300% of the RDA for older men [4].

The mechanism of vitamin B6 toxicity is not fully understood currently; however, recent studies have offered valuable information. In a 2017 study, Vrolijk et al. showed that vitamin B6 toxicity is related to which vitamer is found in excess. Vitamin B6 constitutes a group of six vitamers: pyridoxine (PN), pyridoxal (PL), and pyridoxamine (PM) along with their 5'-phosphate esters (PNP, PLP, and PMP) [1]. PLP is the biologically active form while PN is the inactive form, which is most often used in supplements. The study suggests that the inactive PN can build up when taken in high doses and competitively inhibit the active PLP, causing peripheral neuropathy. In contrast, other inactive vitamers, such as PM, did not affect cellular viability [5].

In a follow-up study, Vrolijk et al. concluded that there are clear inter-individual differences in the metabolism of PN, predisposing some to be sensitive to toxicity [6]. This could begin to explain why this patient experienced symptoms with a daily dose of 6 mg while vitamin B6 has previously been reported as safe in doses up to 500 mg [3]. Additionally, in a 2021 paper, Hadtstein and Vrolijk hypothesized that daily use for an extended period of time can alter the normally rapid excretion of PN, causing an accumulation in the system [7]. This hypothesis would be supported by the presentation seen in this patient where a relatively small daily dose taken for more than 10 years resulted in a clinically significant toxicity.

Peripheral neuropathy due to vitamin B6 toxicity in humans was first reported in the early 1980s. These reports, however, occurred in the context of supplemental vitamin B6 mega-doses of up to 6 g/day for extended periods [2]. Although there is little evidence of their efficacy, mega-doses of 50-500 mg/day were used empirically in the treatment of carpal tunnel syndrome, premenstrual syndrome, and depression, among others [8]. Using these early studies, the US Institute of Medicine set a Tolerable Upper Intake Level (UL) of 100 mg/day in 1998 [1].

However, more recent studies have found evidence of vitamin B6 toxicity in the absence of purposeful mega-doses [9]. Tynes et al. described a 30-year-old post-bariatric surgery patient who developed peripheral neuropathy secondary to vitamin B6 toxicity with their only reported intake being a daily multivitamin containing 2 mg of vitamin B6 and two or three daily energy drinks which contained 10-15 mg vitamin B6. Cessation of multivitamins and energy drinks led to a complete reversal of the patient's symptoms [4]. In 2023, the European Food Safety Administration (EFSA) was requested to deliver a scientific opinion on the safety of vitamin B6, which led to the establishment of a new UL of 12 mg/day [10].

Similar to the case reported by Tynes et al., the peripheral neuropathy with elevated serum vitamin B6 seen in our patient occurred in the context of relatively low daily intake. While retrospective dietary information over the span of three years is difficult to collect, the only reported vitamin B6 supplementation in this patient was in the form of a daily multivitamin containing 6 mg of pyridoxine. With dietary intake in the US at approximately 2 mg/day for men, this would suggest an even lower daily intake than the newly established EFSA UL of 12 mg/day.

## Conclusions

Although significant additional intake from unreported or unknown sources cannot be ruled out, this patient’s presentation suggests the need for more research into the effects of long-term, low-dose pyridoxine supplementation. Additionally, the case highlights the importance of obtaining a complete history of over-the-counter supplement use by primary care physicians. This case also serves as a reminder to keep vitamin B6 toxicity in the differentials for peripheral neuropathy, especially if initial laboratory evaluations are inconclusive. As with most patients with peripheral neuropathy due to vitamin B6 toxicity, this patient is expected to eventually have complete resolution of his symptoms with a return to normal serum vitamin B6 levels.
